# Intrafamilial Patterns of Oral Health: A Cross-Sectional Study of Dental Status Associations Among Children, Parents, and Siblings

**DOI:** 10.3390/jcm14196776

**Published:** 2025-09-25

**Authors:** Zuzanna Borawska, Kinga Wnorowska, Kamila Suchodolska, Justyna Magdalena Hermanowicz, Joanna Bagińska, Magdalena Nowosielska

**Affiliations:** 1 University Hospitals Coventry and Warwickshire Trust, Clifford Bridge Road, Walsgrave, Coventry CV2 2DX, UK; zuzanna.borawska@outlook.com; 2 Dental Surgery “DENTAL PLUS Stomatologia”, ul. Rakowska 6/U2, 02-237 Warszawa, Poland; kingazosiuk@gmail.com; 3 Dental Surgery “DEMED STOMATOLOGIA”, ul. Sybirakow 20/lok 8a, 18-400 Łomża, Poland; kamiladziewiecka@gmail.com; 4 Department of Clinical Pharmacology, Medical University of Bialystok, ul. Mickiewicza 2A, 15-222 Bialystok, Poland; justyna.hermanowicz@umb.edu.pl; 5 Department of Dentistry Propaedeutics, Medical University of Bialystok, ul. Szpitalna 30, 15-295 Bialystok, Poland; joanna.baginska@umb.edu.pl; 6 Department of Gerostomatology, Medical University of Bialystok, ul. Akademicka 3, 15-267 Bialystok, Poland

**Keywords:** caries, determinants of oral health, oral diseases, family environment, Decayed-Missing-Filled Teeth (DMFT) index, DMFT, Dental Treatment Index (DTI)

## Abstract

**Background/Objectives**: Untreated dental caries, the single most common health condition globally, is strongly associated with behavioural factors. This study examined dental status and oral health habits in child–parent and sibling pairs. **Methods**: We retrospectively analysed records from a dental practice in northeastern Poland, including 90 child–parent pairs and 27 sibling pairs. Dental status was assessed using the Decayed-Missing-Filled Teeth (DMFT) index, and treatment completion was measured with the Dental Treatment Index (DTI). Oral health behaviours were also evaluated. **Results**: Significant differences between children and parents were observed only in the mild-to-moderate caries groups (DMFT I: 27 children vs. 12 parents; DMFT II: 15 children vs. 32 parents). No differences were found in the severe caries or caries-free groups. Children had lower treatment completion than parents in the poorest care group (DTI 1: 20 children vs. 7 parents), but similar outcomes in higher DTI categories. Among siblings, differences appeared only in the DMFT I group, with no differences in treatment completion or behaviours. **Conclusions**: Strong similarities in extreme dental characteristics between children and parents, comparable DTI values in most groups, and consistent sibling outcomes suggest that family environment strongly influences oral health.

## 1. Introduction

Oral diseases are a major global health concern, with dental caries being one of the most common non-communicable diseases alongside diabetes and cardiovascular disease [1,2,3]. Socio-behavioural and environmental factors play crucial roles in disease development, accounting for approximately 40% of deaths annually in the US [2,3,4]. In response, the World Health Organization (WHO) has emphasised “Healthy Living” policies, highlighting the influence of social and community contexts on health outcomes [5,6].

Understanding how these health patterns develop requires examining their origins in family environments. Health behaviours are shaped primarily within the family. They are acquired through observation, imitation, and shared daily routines, a process often referred to as “community learning” [7,8,9]. Families provide the strongest environment for this learning because of emotional bonds, shared time, unlimited behavioural observation, and repetitive exposure [10]. These patterns are effectively “transmitted” across generations, much like genetic traits [11,12,13,14,15].

This family influence is especially visible in oral health, where caries reflects both biological and social determinants. Key factors influencing children’s oral health outcomes include socioeconomic status, parental education, family affluence, and access to preventive services [16,17,18]. Early social conditions establish oral health trajectories that persist over time, reinforcing inequalities in diet and access to treatment through structural barriers such as healthcare financing and community resources [19,20]. These persistent inequalities are driven by upstream social gradients and have been further exacerbated by global challenges such as the COVID-19 syndemic [5,6,7,21].

Beyond these structural determinants, psychological aspects of oral health also show intrafamilial transmission. Studies have found associations between parental dental anxiety and children’s dental fear, phobia symptoms, and oral health outcomes [22,23]. Poor parental knowledge has similarly been linked to higher dental anxiety in children after dental trauma [24]. Together, these findings indicate that both cognitive and emotional dimensions of oral health can be transmitted within families.

The universality of family influence on oral health, however, manifests differently across healthcare systems and cultural contexts. International research across diverse healthcare systems reveals significant cross-cultural variations in intrafamilial oral health patterns and anxiety transmission. A comprehensive six-country European study of 731 families from the Balkans and Central Europe found regional differences in the prevalence of dental anxiety among children, with children’s high anxiety levels ranging from 10.94% in Bosnia and Herzegovina to 26.67% in North Macedonia, though the correlation between parents and children remained consistent (r = 0.4, *p* < 0.01) [25]. Such variation suggests that healthcare system design and cultural family dynamics modify the way biological and behavioural transmission mechanisms are expressed. For example, Nordic countries provide comprehensive public dental care for children, reducing family-level disparities compared with mixed public–private systems more common in Central and Southern Europe [26,27,28]. These international comparisons underline that while family environment is a universal determinant of oral health, its impact is shaped by system- and culture-specific factors [29].

Building on this background, the aim of this study was to examine associations between family environment and oral health outcomes in children. Specifically, we compared the following variables: (1) dental status, measured by the Decayed-Missing-Filled Teeth (DMFT) index, and treatment completion, measured by the Dental Treatment Index (DTI), between children and their parents and between siblings; and (2) oral health behaviour patterns between siblings.

We tested the null hypothesis that there are no statistically significant differences in dental status and behavioural habits between children and their parents, or between siblings.

## 2. Materials and Methods

### 2.1. Study Design and Setting

We conducted a retrospective cross-sectional study using dental records collected between 2017 and 2019 from a single dental practice in northeastern Poland. The clinic serves both urban (67%) and rural (33%) populations. The practice follows standard Polish dental care protocols and serves families across diverse socioeconomic backgrounds typical of the regional population. The study was approved by the Biomedical Committee of the Medical University of Bialystok (resolution nr R-I-002/36/2019).

### 2.2. Study Population

From 203 paediatric patient records, we identified 90 children whose parents also had complete treatment documentation at the same practice. When both parents met the inclusion criteria, the parent with the greater number of documented visits was selected. This process yielded 90 child–parent pairs for analysis.

We also identified 27 sibling pairs who both received treatment at the clinic and had complete dental documentation. Subjects with incomplete records were excluded. Detailed inclusion and exclusion criteria, along with sample size calculation, are presented in Appendix A.

### 2.3. Dental Status Assessment

Dental status was evaluated using the Decayed-Missing-Filled Teeth (DMFT) index. To allow comparisons across age groups with different numbers of teeth, DMFT values were converted to percentages (see Appendix A for details). Clinical examinations were performed by a calibrated dentist following WHO criteria (see Appendix A for calibration details), under artificial lighting, using dental mirrors and probes.

Treatment completion was assessed with the Dental Treatment Index (DTI), calculated for individuals with DMFT > 0 as the ratio of filled teeth to the sum of decayed and filled teeth. A higher DTI indicates more effective treatment (see Appendix A).

### 2.4. Group Classification

Participants were categorised into subgroups based on caries severity (DMFT 0–IV) and treatment completion (DTI 1–4), as shown in Table 1. For sibling pairs, we also compared oral health behaviours, including toothbrush type, brushing frequency, dental visit attendance, and sweet consumption. Behavioural data were obtained from routine questionnaires completed at the first visit and updated every six months.

### 2.5. Statistical Analysis

Associations between categorical variables were tested using the chi-square independence test. For paired variables, McNemar’s test was applied. Analyses were performed with GraphPad Prism 9.4 software (Boston, MA, USA), and statistical significance was set at *p* < 0.05.

## 3. Results

### 3.1. Demographics

**Figure 1:**
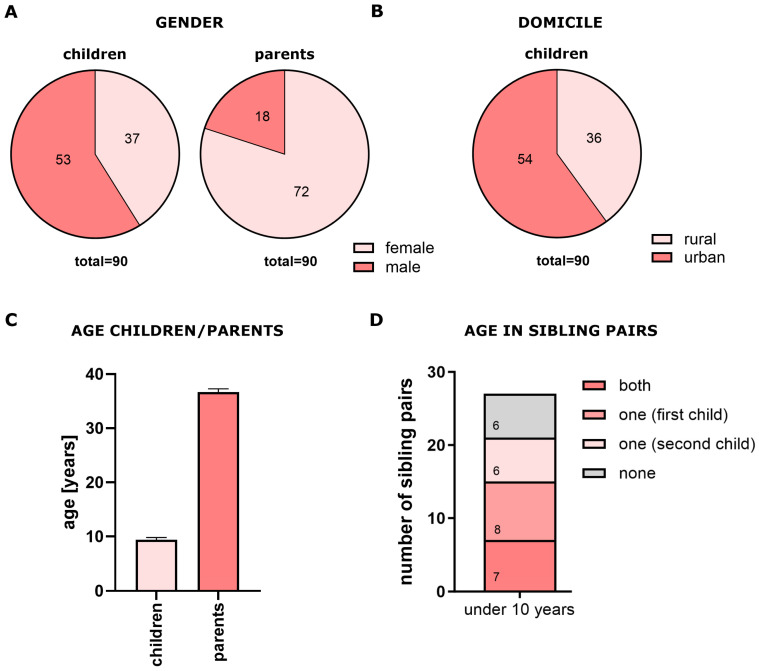
Demographic characteristics of study population. (**A**) Sex distribution of children and parents; (**B**) urban–rural distribution; (**C**) age distribution of children and parents (mean ± SEM); (**D**) age distribution of siblings.

The subjects of the study included 90 children (53 boys, 37 girls) and their parents, most of whom were mothers (Figure 1A). We also identified 27 sibling pairs. Figure 1B shows the urban-rural distribution of participants, while Figure 1C presents the age distributions for children and parents (children: 9.3 ± 0.4 years; parents: 36.6 ± 5.7 years, mean ± SEM, where SEM = standard error of the mean). No significant age differences were observed between siblings (Figure 1D). For subgroup analysis, children were divided according to whether they were younger or older than 10 years of age (χ^2^ = 0.071, *p* = ns, OR = 0.75, 95% CI: 0.214–2.465).

### 3.2. Parent–Child Comparisons

We compared dental status and treatment completion between children and their parents using paired analysis. Participants were classified into five DMFT subgroups and four DTI subgroups (Table 1).

Of the 90 child–parent pairs, 81 had DMFT > 0. No pair had both parent and child with DMFT = 0, though this difference was not statistically significant.

DMFT Analysis: Significant differences between parents and children were observed only in the intermediate categories:

- DMFT I (mild caries, 1–25% teeth affected): OR = 0.44 (95% CI: 0.21–0.91, *p* < 0.05; Table 2);
- DMFT II (moderate caries, 26–50% teeth affected): OR = 2.13 (95% CI: 1.12–4.24, *p* < 0.05; Table 2).

**Table 2:** Distribution and statistical analysis of Decayed-Missing-Filled Teeth (DMFT) and Decayed Teeth Index (DTI) groups in parent–child pairs. ns = not significant. *p* < 0.05 (chi-square test). * denotes statistically significant difference.

	Chi-Square		95% Confidence		
Decayed-Missing-Filled Teeth	Value	*p*-Value	Odds Ratio	Lower	Upper
DMFT 0	1.78	*p* = ns	0.29	0.03	1.50
DMFT I	5.03	* *p* < 0.05	0.44	0.21	0.91
DMFT II	5.45	* *p* < 0.05	2.13	1.12	4.24
DMFT III	1.44	*p* = ns	1.62	0.77	3.51
DMFT IV	0.75	*p* = ns	0.50	0.11	1.87
**Dental Treatment Index**					
DTI 1	5.33	* *p* < 0.05	0.35	0.13	0.86
DTI 2	0.00	*p* = ns	0.94	0.43	2.03
DTI 3	1.63	*p* = ns	0.58	0.25	1.28
DTI 4	1.24	*p* = ns	0.61	0.26	1.37

**Figure 2:**
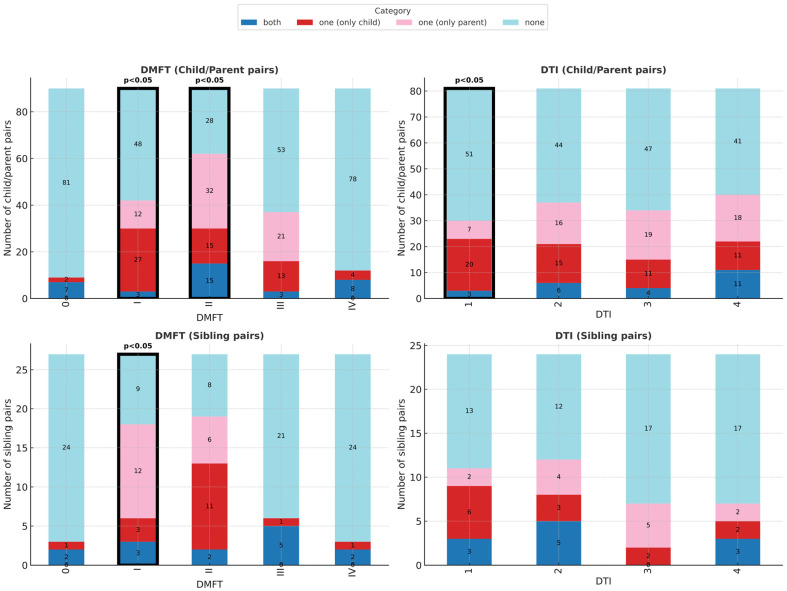
Dental status and treatment outcomes in child–parent and sibling pairs. Distribution across DMFT (caries severity) and DTI (treatment completion) subgroups. Significant differences were found in DMFT I and II (child–parent pairs), DTI 1 (child–parent pairs), and DMFT I (sibling pairs).

No differences were observed in the extreme categories (caries-free, severe, or very severe caries groups), suggesting strong parent–child concordance in these groups (Figure 2).

DTI Analysis: DTI was calculated for 81 pairs (excluding seven children and two parents with DMFT = 0). Children had significantly lower treatment completion than parents only in the poorest care category (DTI 1: OR = 0.35, 95% CI: 0.13–0.86, *p* < 0.05; Table 3). In higher categories (DTI 2–4), outcomes were comparable (Figure 2).

### 3.3. Sibling Comparisons

**Figure 3:**
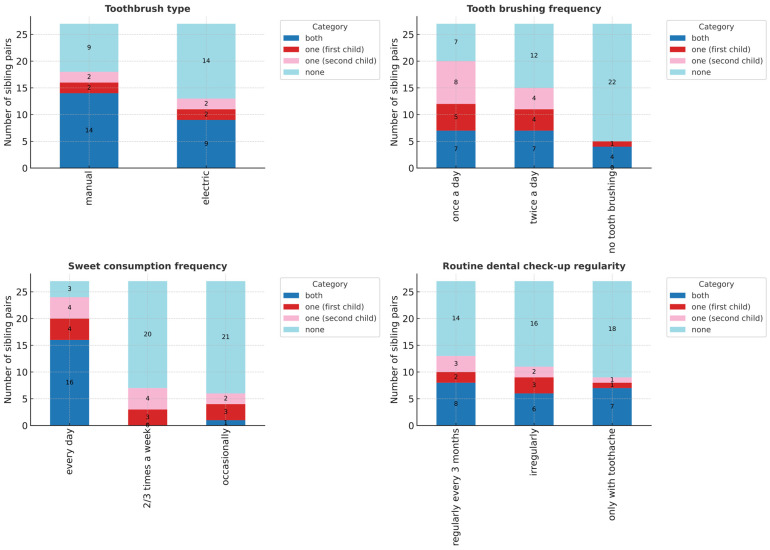
Oral health behaviours in sibling pairs. Comparison of toothbrush type, brushing frequency, dental attendance, and sweet consumption. No statistically significant differences were observed.

Analysis of sibling pairs (n = 27, limited statistical power) revealed significant differences only in the mild caries group (DMFT I: OR = 4.0, 95% CI: 1.08–22.09, *p* < 0.05; Table 3). No differences were found in treatment completion (DTI) or in oral health behaviours such as toothbrush type, brushing frequency, dental visits, or sweet consumption (Figure 3).

Overall, siblings demonstrated high behavioural consistency, with individual variation appearing only in early caries development.

## 4. Discussion

The public health principle “health begins at home” highlights the central role of families in shaping health behaviours. Lifestyle—defined as the sum of health-promoting and health-damaging behaviours learned in childhood and carried into adulthood—is among the strongest determinants of health and wellbeing [18,30,31,32,33]. Dental caries, like other chronic diseases such as diabetes or cardiovascular disease, is largely driven by non-medical factors, with around 80% of its risk attributable to behavioural and environmental influences [3,19,21,32].

### 4.1. Parent–Child Relationships in Oral Health

Our findings show clear parent–child similarities in dental health. Mothers predominantly accompanied children to dental visits (Figure 1), reflecting their dominant role in modelling health behaviours [33,34]. Mothers may be best positioned to monitor disease progression and treatment management, as well as treatment guidelines and recommendations for their children. This highlights mothers as key targets for prevention and education programs.

Strong concordance appeared in extreme categories: families were either consistently caries-free or severely affected (DMFT III–IV). In contrast, mild-to-moderate categories (DMFT I–II) showed greater variation, suggesting that families are most similar when disease burden is very low or very high, but more heterogeneous in intermediate states. Differences in these groups may reflect biological susceptibility or parental underestimation of early disease in children. Strong concordance in extreme categories (caries-free and severe disease) reflects consistent family behavioural patterns—families with excellent hygiene maintain health across members, while families with poor habits show universal disease burden.

### 4.2. Quality Disparities

The DTI analysis revealed concerning disparities in treatment quality. Group DTI 1 (poorest treatment completion) showed the most significant parent–child differences, with children receiving inferior care in 20 pairs compared to their parents (Figure 2). This is particularly troubling given that caries progresses most rapidly during childhood. Clinicians notice that most dental interventions concern teeth initially treated during childhood and adolescence (secondary or atypical caries). The superior parental dental status in this group suggests these parents benefited from better childhood dental care but failed to provide equivalent care for their children.

One possible explanation is structural. Until recently, school-based dental programs provided universal care for Polish children, but these services were discontinued. Parents may have benefited from school-based care in their youth, while today’s children must rely on less accessible clinic-based services. It could be inferred that this change in public health strategy has acted to the disadvantage of children, contributing to inferior dental status compared to their parents.

Other factors, such as limited provider comfort with young patients or scheduling difficulties, may also contribute, even though financial coverage for paediatric care is provided by the National Health Fund.

### 4.3. Sibling Comparisons and Family Environment

Sibling analysis, though limited by sample size, demonstrated striking behavioural concordance. Brushing habits, dental attendance, and sweet consumption were nearly identical within families (Figure 3). This supports the strong role of family environment in shaping oral health behaviours. Children observe and replicate behaviours within their family unit, particularly from parents and older siblings [35].

Despite behavioural similarities, some variation emerged in early disease expression (DMFT I). This likely reflects individual biological differences in susceptibility or parental attention. Importantly, the absence of differences in treatment completion (DTI) suggests that once caries is diagnosed, families provide similar treatment access to all children.

### 4.4. International Context

Our findings need to be considered in light of healthcare system differences. The Polish healthcare system presents a particularly instructive case study, representing a mixed public–private model characteristic of post-transition economies in Central Europe. In Poland, children are entitled to free dental care until age 18, but oral health accounts for only 2.7% of public healthcare spending, with state-financed oral services utilised by approximately 30% of children and adolescents and just 15% of adults [36]. This limited public coverage, combined with basic dental materials being the only state-funded options, creates substantial reliance on private care for comprehensive treatment. This leads to significant socioeconomic disparities: 92% of Polish teenagers experience caries, and rural children often lack access to public services [37].

Such healthcare infrastructure characteristics may amplify intrafamilial oral health clustering in several ways. In mixed public–private systems such as Poland’s, treatment choices may be shaped by financial considerations, intensifying parental influence on children’s oral health outcomes. This contrasts with universal public models, such as those in Nordic countries, where family-level socioeconomic differences are less likely to determine treatment access [26,27,28]. Such comparisons highlight the importance of tailoring prevention strategies to healthcare financing structures and cultural contexts.

### 4.5. Clinical and Public Health Implications

These findings challenge models that attribute caries solely to infectious transmission, instead emphasizing the importance of lifestyle, behaviour, and education [38,39]. Families emerge as critical units for interventions, as summarised in Table 4.

Routine family-based screening should be considered: when one family member has extreme oral health status, other members should be assessed and appropriate preventive or therapeutic measures provided. Prevention efforts should target high-risk families where parents have poor dental status or low treatment completion, since children in these households are most vulnerable.

Public health campaigns should also raise awareness of the importance of early dental visits. Regular dental attendance from early childhood significantly impacts lifelong oral health [40]. However, while parents often attend medical appointments for young children, they may neglect dental care in early childhood [41,42]. Strengthening awareness of dentists’ role in paediatric health could improve long-term outcomes.

### 4.6. Study Limitations and Strengths

Limitations. Our study has several important limitations. The retrospective cross-sectional design prevents establishing causal relationships between parental dental status and children’s outcomes or determining whether shared behaviours cause similar outcomes. Significant unmeasured confounders include genetics, environmental factors (water quality, pollution), and, most importantly, socioeconomic status. Socioeconomic status is particularly important, as it influences oral health through multiple pathways: direct effects via access to preventive care, healthy foods, and quality oral hygiene products; indirect effects through health literacy, parental supervision time, and stress levels affecting health prioritisation; and environmental effects, including neighbourhood water fluoridation, local food environments, and school-based prevention programs. Our observed family concordance may largely reflect shared socioeconomic circumstances rather than direct behavioural transmission. In future research, we could stratify families by their household income to remove its confounding effect.

The single-practice design and modest sample size (90 child–parent pairs, 27 sibling pairs) limit generalisability and statistical power, especially for subgroup analyses. It also introduces selection bias, as families may share similar socioeconomic characteristics and healthcare-seeking behaviours. Families with poor oral health or irregular attendance may also be underrepresented (survival bias), potentially leading our sample to over-represent families with better oral health outcomes and more consistent healthcare utilisation.

Strengths. Despite these limitations, our study provides valuable evidence for the underestimated role of family environment in oral health outcomes through an innovative intrafamilial design that addresses a significant gap in the literature.

Strengths include the intrafamilial design, which allowed direct comparisons between related individuals, the use of objective clinical indices (DMFT, DTI), and the inclusion of both parent–child and sibling analyses. Using medical records rather than self-reports adds reliability, and the dual-relationship design offers insights into both intergenerational and household-level influences.

To our knowledge, this represents one of the first studies to conduct systematic comparative analysis of dental status among multiple family members across different age groups using standardised clinical measures. Existing literature primarily examines associations between children’s oral health and parental sociodemographic characteristics (education, residence, socioeconomic status) rather than direct intrafamilial clinical concordance. Our direct comparison approach between related individuals provides new insights into family-level oral health patterns and offers a foundation for developing family-centred prevention strategies.

### 4.7. Conclusions and Future Directions

Our study highlights the strong influence of family environment on oral health. Key findings include

1. Selective concordance between parents and children in mild-to-moderate caries, with strong similarities in extreme categories (caries-free and severe disease).
2. Treatment disparities, with children receiving less complete care than parents in families with the poorest oral health.
3. Behavioural consistency among siblings, indicating powerful family-level influence, despite individual differences in early disease expression.
4. Support for family-centred strategies, suggesting that prevention and screening should target entire households rather than individuals.

These results challenge traditional individual-focused care models and argue for family-based screening protocols, especially in mixed public–private healthcare systems where socioeconomic status strongly affects treatment access and quality.

Future research should expand to multi-centre samples and include socioeconomic stratification to account for confounding effects. Advanced analytical approaches, such as clustering or similarity-based methods, may help identify distinct family oral health profiles. By doing so, we can design more effective prevention strategies that address both intergenerational and household-level influences.

## Figures and Tables

**Table 1:** Criteria and characteristics for Decayed-Missing-Filled Teeth (DMFT) index and Dental Treatment Index (DTI) subgroups.

Subgroup	Subgroup Criteria	Caries Severity/Treatment Completion
DMFT 0	No caries	Caries-free
DMFT I	Caries affecting 1–25% of teeth	Mild caries
DMFT II	Caries affecting 26–50% of teeth	Moderate caries
DMFT III	Caries affecting 51–75% of teeth	Severe caries
DMFT IV	Caries affecting 76–100% of teeth	Very severe caries
DTI 1	<25% of carious teeth have been treated	Very poor treatment completion
DTI 2	26–50% of carious teeth have been treated	Poor treatment completion
DTI 3	51–75% of carious teeth have been treated	Moderate treatment completion
DTI 4	76–100% of carious teeth have been treated	Good treatment completion

**Table 3:** Distribution and statistical analysis of Decayed-Missing-Filled Teeth (DMFT), Decayed Teeth Index (DTI) and oral health behaviours in sibling pairs. ns = not significant. *p* < 0.05 (chi-square test). * denotes statistically significant difference.

	Chi-Square			95% Confidence	
Decayed, Missing, Filled Teeth Index	Value	*p*-Value	Odds Ratio	Lower	Upper
DMFT 0	0.00	*p* = ns	0.50	0.01	9.61
DMFT I	4.27	* *p* < 0.05	4.00	1.08	22.09
DMFT II	0.94	*p* = ns	0.55	0.17	1.61
DMFT III	1.50	*p* = ns	0.20	0.00	1.79
DMFT IV	0.00	*p* = ns	0.50	0.01	9.61
**Dental Treatment Index**					
DTI 1	1.13	*p* = ns	0.33	0.03	1.86
DTI 2	0.00	*p* = ns	1.33	0.23	9.10
DTI 3	0.57	*p* = ns	2.50	0.41	26.25
DTI 4	0.25	*p* = ns	1.00	0.07	13.80
**Type of toothbrush**					
Manual	0.25	*p* = ns	1.00	0.07	13.80
Electric	0.25	*p* = ns	1.00	0.07	13.80
**Tooth brushing frequency**					
Once a day	0.31	*p* = ns	1.60	0.46	6.22
Twice a day	0.13	*p* = ns	1.00	0.19	5.37
Does not brush teeth every day	0.31	*p* = ns	0.25	0.01	2.53
**Frequency of eating sweets**					
Every day	0.13	*p* = ns	1.00	0.19	5.37
2 or 3 times a week	0.00	*p* = ns	1.33	0.23	0.10
Occasionally	0.00	*p* = ns	0.67	0.06	5.82
Dental check-ups					
Regularly every 3 months	0.00	*p* = ns	1.50	0.17	17.96
Less regularly than every 3 months	0.00	*p* = ns	0.67	0.06	5.82
Only symptomatic	0.50	*p* = ns	1.00	0.01	78.50

**Table 4:** Summary of study findings, results, conclusions, and clinical significance.

Finding	Statistical Result	Conclusion	Clinical Significance
Parent–child concordance in extreme categories	No significant differences in DMFT 0, III, IV (*p* > 0.05)	Families cluster at extremes of health and disease	Family-based screening: Screen all family members when one has extreme oral health; universal prevention for healthy families, targeted interventions for severely affected families
Parent–child differences in moderate disease	Significant differences in DMFT I (OR = 0.44, *p* < 0.05) and DMFT II (OR = 2.13, *p* < 0.05)	Parents may underestimate children’s early caries	Enhanced early detection: Strengthen parental education on early detection and treatment
Paediatric treatment disadvantage	Children had poorer DTI in group 1 (OR = 0.35, *p* < 0.05). 20/90 pairs affected	Children receive less complete care than parents in high-risk families	Healthcare equity advocacy: Prioritise paediatric care in families with poor oral health
Sibling behavioural concordance	No significant differences in health behaviours between siblings (*p* > 0.05 for all behaviours)	Siblings adopt nearly identical behaviours	Family-centred interventions: Family-wide prevention strategies are efficient and effective
Limited sibling clinical differences	Significant difference only in DMFT I (OR = 4.0, *p* < 0.05)	Individual susceptibility influences early caries	Individualized assessment within families: Monitor each child individually despite shared behaviours

## Data Availability

The data presented in this study are available on request from the corresponding author (the data are not publicly available due to privacy or ethical restrictions).

## Abbreviations

The following abbreviations are used in this manuscript:

DMFT	Decayed Missed Filled Teeth
DTI	Dental Treatment Index
SEM	Standard Error of the Mean

## Appendix A. Materials and Methods

### Appendix A.1. Sample Size Calculation

The minimum sample size was determined based on population data from the most recent Polish oral health monitoring report [43]. Using a population proportion of 90% derived from this national survey data, the minimum required sample size was calculated to be 83. This calculation ensures adequate statistical power to detect meaningful differences in oral health outcomes within the study population while maintaining appropriate precision for the estimated proportions.

### Appendix A.2. DMFT Index Conversion to Percentage

To assess the dental condition, an original method was used that enables comparison of caries experience between participants of different ages. To obtain comparable values, for each child and parent, the percentage of teeth affected by caries disease was determined, expressed as DMFT + dmft/X. X represents the number of teeth determined as follows:

- In the children’s group, X was taken as the sum of the number of primary and permanent teeth present in the oral cavity plus the number of teeth extracted due to caries (MT/mt) if such extractions were performed. Primary teeth lost due to physiological tooth exfoliation, unerupted primary and permanent teeth, teeth lost due to trauma, and teeth removed for orthodontic reasons were not counted as MT/mt.
- In the adult group, X was taken as the sum of the number of permanent teeth present in the oral cavity, including third molars, plus the number of teeth extracted due to caries (MT), if such extractions were performed. Unerupted teeth, teeth lost due to trauma or periodontal disease, and teeth removed for orthodontic reasons were not counted as MT.

### Appendix A.3. DMFT Examiner Calibration

The examination was conducted by one researcher (KS) according to WHO recommendations and based on the criteria for clinical condition classification (Oral Health Surveys. Basic Data. WHO Geneva 1997). The researcher was trained by a member of the epidemiological team of calibrated researchers (MN) in a training and calibration process for the purpose of conducting epidemiological studies titled: “Monitoring the oral health status of the Polish population in 2016–2020” in the northeastern region of Poland [44]. Differences in the two examinations concerned only the determined number of D (decayed teeth). Inter-examiner reliability was assessed using 18 patients examined by both the principal investigator (MN) and trained researcher (KS). Cohen’s kappa coefficient for the decayed teeth component was κ = 0.923. Intra-examiner reliability was evaluated by having researcher KS re-examine the same 18 patients after 10 days, yielding κ = 0.943, indicating excellent agreement for both inter- and intra-examiner reliability.

### Appendix A.4. DTI Calculation

The DTI was determined for permanent dentition using the formula F/D + F, for primary dentition: f/d + f, and for mixed dentition (F + f)/(D + d) + (F + f). Values range from 0 to 1, where 1 means that all teeth with caries were treated, while 0 means that no tooth with caries was treated. The result (being a fraction) was then converted to %. It was determined for individuals with DMFT (or dmft) > 0 [45,46]—methodological references.

**Table A1:** Inclusion and exclusion criteria.

Category	Inclusion Criteria	Exclusion Criteria
General Criteria (All Participants)	Dental examination completed and documented at participating dental practice between 2017 and 2019 (inclusive) Examination performed by the same calibrated examiner Complete examination records, including - DMFT (Decayed, Missing, Filled Teeth) index - DTI (Dental Treatment Index) - Number of teeth extracted due to causes other than caries and its complications	Incomplete dental examination records Examination performed by different or non-calibrated examiners Missing essential data components (DMFT, DTI, or extraction records) Examination date outside 2017–2019 study period
Child–Parent Pairs	Both child and parent individually meet all general inclusion criteria Both examined at the same dental practice during study period When both parents meet criteria, parent with higher number of visits during 2017–2019 selected	Either child or parent fails to meet general inclusion criteria Incomplete data for any family member intended for analysis Examinations conducted at different dental practices
Sibling Pairs	Two siblings both meet general inclusion criteria Both siblings examined at the same dental practice during study period Only sibling pairs included in analysis	Families with more than two children meeting criteria (excluded from sibling analysis) Siblings examined at different dental practices Either sibling fails to meet general inclusion criteria
